# Induced, Imprinted, and Primed Responses to Changing Environments: Does Metabolism Store and Process Information?

**DOI:** 10.3389/fpls.2019.00106

**Published:** 2019-02-13

**Authors:** Jens Schwachtje, Sarah J. Whitcomb, Alexandre Augusto Pereira Firmino, Ellen Zuther, Dirk K. Hincha, Joachim Kopka

**Affiliations:** Department of Molecular Physiology, Applied Metabolome Analysis, Max-Planck-Institute of Molecular Plant Physiology, Potsdam, Germany

**Keywords:** priming, stress response, stress signaling, metabolism, metabolic imprint, plant physiology

## Abstract

Metabolism is the system layer that determines growth by the rate of matter uptake and conversion into biomass. The scaffold of enzymatic reaction rates drives the metabolic network in a given physico-chemical environment. In response to the diverse environmental stresses, plants have evolved the capability of integrating macro- and micro-environmental events to be prepared, i.e., to be primed for upcoming environmental challenges. The hierarchical view on stress signaling, where metabolites are seen as final downstream products, has recently been complemented by findings that metabolites themselves function as stress signals. We present a systematic concept of metabolic responses that are induced by environmental stresses and persist in the plant system. Such metabolic imprints may prime metabolic responses of plants for subsequent environmental stresses. We describe response types with examples of biotic and abiotic environmental stresses and suggest that plants use metabolic imprints, the metabolic changes that last beyond recovery from stress events, and priming, the imprints that function to prepare for upcoming stresses, to integrate diverse environmental stress histories. As a consequence, even genetically identical plants should be studied and understood as phenotypically plastic organisms that continuously adjust their metabolic state in response to their individually experienced local environment. To explore the occurrence and to unravel functions of metabolic imprints, we encourage researchers to extend stress studies by including detailed metabolic and stress response monitoring into extended recovery phases.

## Introduction

Sessile plants are forced to respond to adverse biotic and abiotic conditions in their local environment. Depending on the nature and intensity of such conditions, a plant’s physiology can change markedly, generally because of stress-activated signaling that leads to specific physiological responses. These responses protect against or mitigate deleterious effects of stress. In a top-to-bottom view, stress-related cues induce signaling cascades, followed by activities at genetic and protein levels. Metabolite changes are generally considered to be the last step in this event cascade. Typically, large parts of metabolism are affected. Stress-related cues may directly influence enzyme activities that modify the metabolic state, independent of the transcription/translation machinery. Finally, metabolic changes result from cues that do not induce classical stress-signaling. Such direct changes of metabolism are caused by the fluctuating physico-chemical environment of a plant, such as varying climate parameters, e.g., temperature, or soil properties (Hawkes and Sullivan, 2001; De Deyn et al., 2008; Sampaio et al., 2015; Onwuka and Mang, 2018).

Most environmental stresses are transient, such as temporally limited temperature extremes or drought phases, insect attacks or microbial infections. After a stress has ended, plants may recover from the stress and reset metabolism to growth and reproduction modes; however, recovery may not be complete. Even short-term environmental stress may have long-lasting effects on the plant system. A growing number of studies suggest that storing information about a past stress event benefits plants by preparing them for the same or a similar stress in the future. This phenomenon is called priming (Frost et al., 2008; Gamir et al., 2014a; Conrath et al., 2015; Hilker et al., 2015; Mauch-Mani et al., 2017). Several plant priming mechanisms are well investigated (Table 1).

**Table 1:** Examples of stress priming scenarios. Many abiotic and biotic stresses lead to imprints that improve the plant’s response to a subsequent stress.

Priming stress	Induction by	Reference
Biotic/insect	Feeding	Frost et al., 2008
	Oviposition	Bandoly et al., 2016
	Volatiles	Engelberth et al., 2004
Biotic/microorganism	Pathogens (SAR)	Jaskiewicz et al., 2011; Dempsey and Klessig, 2012; Zeier, 2013
	Rhizobacteria (ISR)	van Loon, 2007; van der Ent et al., 2009
	Symbiotic fungi	Pozo et al., 2009
Abiotic	Cold/freezing	Thomashow, 1999; Guy, 2003; Hincha and Zuther, 2014; Zuther et al., 2018
	Salt	Sani et al., 2013
	Heat	Stief et al., 2014; Bäurle, 2016
	Drought	Ding et al., 2012
Other stimuli	β-Aminobutyric acid	Zimmerli et al., 2001
	Salicylic acid	Jakab et al., 2001
	Seed priming with different techniques	Jisha et al., 2013; Donohue, 2009; Rasmann et al., 2011

Priming mechanisms are described at different levels: at the epigenetic level (e.g., by DNA and histone modification) and at the transcript or protein level (e.g., persisting changes in the abundance of transcripts, including transcription factors, and proteins or modulation of enzyme activities). However, the metabolic level as a mediator of priming has remained largely unexplored, even though large parts of metabolism are altered during stress (e.g., Schwachtje and Baldwin, 2006; Bolton, 2009; Krasensky and Jonak, 2012; Fraire-Velázquez and Balderas-Hernández, 2013). Here, we hypothesize that persistent stress-induced changes in metabolite concentrations, metabolite ratios, or metabolic fluxes represent a **metabolic imprint** of prior environmental impacts and that these imprints can prime responses to future environmental events. We present evidence that supports our hypothesis and suggest environmental shift experiments that not only monitor metabolic responses during a first stress exposure (the priming event) or during a second stress response (the primed response), but also monitor short- and long-term recovery phases after stress events. Such experimental designs may characterize and identify functions of **metabolic imprints** at the level of **metabolic priming.**

Metabolite-primed responses are only properly defined by the timing, nature, and dose of the preceding environmental change, the duration of the recovery period, and in addition by the nature and dose of the subsequent stress for which metabolism is primed. In agreement with generalized concepts of non-metabolic primed stress responses (Hilker et al., 2015), several scenarios of metabolite-primed stress responses are conceivable:

1. The primed response may be stronger than the non-primed response level. As a result, defense or tolerance mechanisms can be amplified.
2. The primed response rate may be accelerated and reach effective response levels earlier.
3. The primed response is initiated earlier. In this case, the system’s response rate may remain unchanged, but the effective levels of response are reached earlier.
4. Primed responses may be triggered by a lower stress dose.

These scenarios are thought to be generally applicable and have been recently discussed (Hilker et al., 2015). In the following, we focus on the roles of metabolites during stress responses, recovery, and priming. We shortly highlight effects of metabolites at all system levels of plant physiology and subsequently review metabolic changes that are caused by stress and last during stress recovery as metabolic imprints. We link metabolic imprints to a wide range of abiotic and biotic stresses. Finally, we discuss experimental approaches that enable discovery of metabolic imprints and functional analyses of these imprints.

## Induced Metabolic Responses

Changes in the biotic and abiotic environment are reflected by the metabolic state of a plant. Plants have a multitude of plastic responses hardwired into their genomes (Sultan, 2000). These responses are induced concomitantly with the stress and function as defense, tolerance, or repair mechanisms (e.g., Dangl and Jones, 2001; Suzuki et al., 2011; Schuman and Baldwin, 2015). These mechanisms can be defined as **stress**-**signaling dependent metabolic responses.** Additionally, physical and chemical conditions such as temperature and soil nutrients influence metabolism, albeit mostly to a lesser degree than stresses. Temperature affects all reaction and transport rates. Soil nutrients influence physiology according to their availability. Consequently, changes in the physico-chemical environment of a plant will cause concomitant metabolic responses which influence single or multiple nutrient fluxes through the plant system. These changes may ultimately become apparent as changes in metabolic pool sizes or fluxes. Therefore, **induced metabolic responses** need to be interpreted as the synergistic effects of stress-signaling dependent plant responses and of external physico-chemical influences.

**Figure 1:**
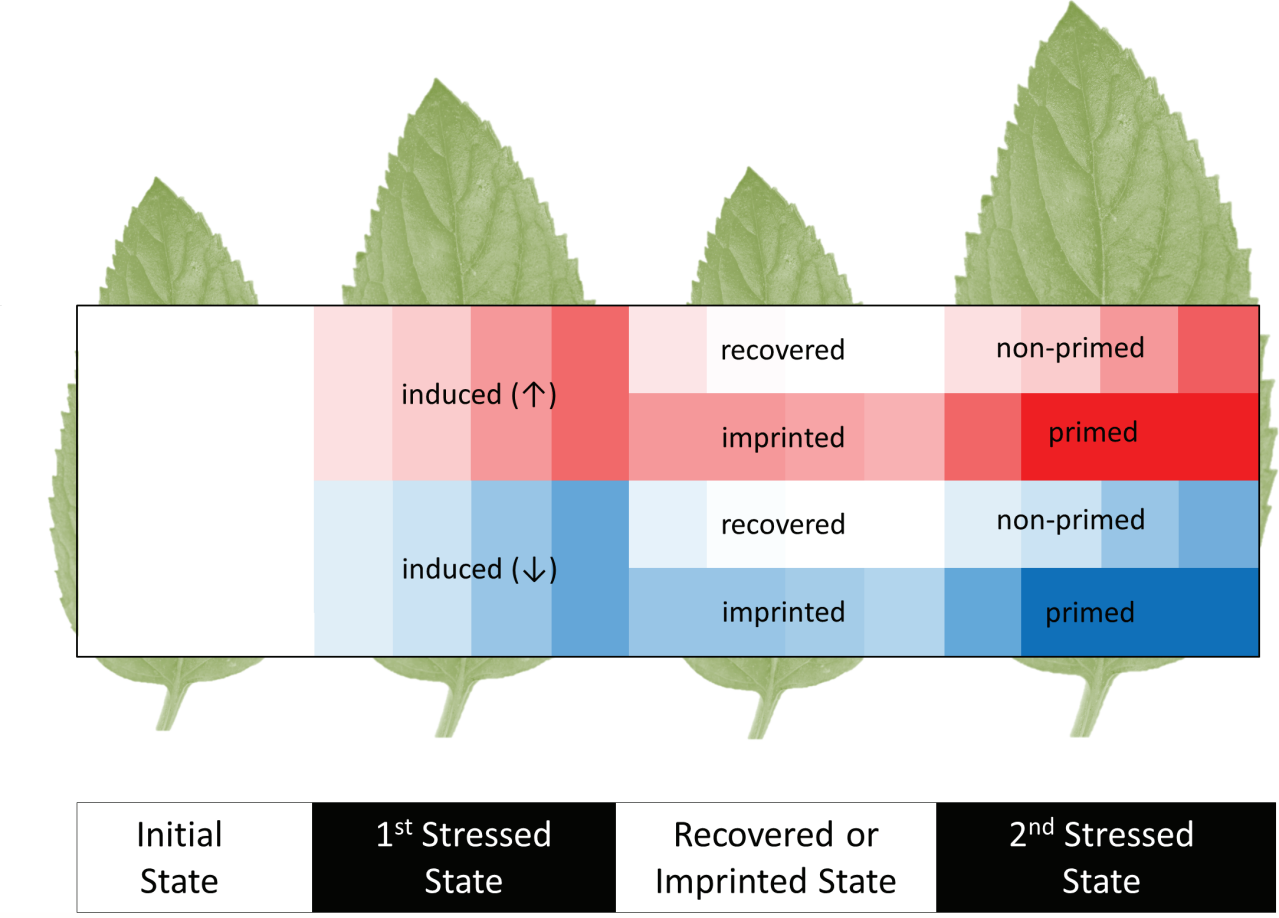
Scheme of initial, induced, imprinted, and primed metabolic responses to recurrent abiotic or biotic stresses. The heat map shows increases (↑, red) and decreases (↓, blue) of metabolite levels relative to the pre-stressed, initial metabolic state (white). Most induced responses that are caused by a first stress period subsequently recover to the initial metabolic states. Few metabolic changes are retained. We argue that these metabolic imprints may be used as a memory that stores and processes information of preceding stress histories. These processes may contribute to stress priming. A primed response to a second stress enables organisms to cope better with recurrent stresses. Parts of the primed metabolic responses can be more sensitive, earlier, faster, or result in more extreme changes of metabolite levels. Note that imprinted metabolites may not necessarily have primed responses. Indeed, metabolic imprints during recovery without prior induction and primed responses without prior metabolic induction or imprinting are conceivable.

The study of induced metabolic responses implies an ***initial metabolic state*** that transitions into an ***induced metabolic state*** (Figure 1); however, these states cannot be viewed as steady states, because they are integrated into non-static physiological processes governed by diurnal environmental cycles, circadian rhythms, and developmental progression of specific tissues and of the whole plant system. Due to these interactions, any observed induced metabolic response may represent a direct modification of metabolism that is on top of the underlying physiological programs of the plant.

## Metabolic Imprints and Metabolic Memory

After an environmental stress has ceased, plant metabolism typically returns to a recovered state that is highly similar to the initial state (e.g., Hemme et al., 2014; Crisp et al., 2016; Pagter et al., 2017). A very basic metabolic perturbation and recovery process may resemble a hysteresis curve, where the metabolic transition during the perturbation and recovery take characteristically different paths. The kinetics of metabolic remodeling are largely dependent on the nature of the environmental stress, transcriptional activities, the architecture of the affected metabolic networks, and the set of transport rates and enzyme activities that act on the induced metabolite levels. As an example of differential metabolic remodeling kinetics, glycolysis intermediates reached pre-stress levels quicker than TCA cycle intermediates in *Arabidopsis* roots upon recovery from oxidative stress (Lehmann et al., 2012).

Some induced metabolic responses may persist after the global metabolic state of the plant has recovered to the initial state (Figure 1). A simple case would be delayed adjustment of a metabolite to the initial state. Such a delay causes a metabolite to be more abundant at the onset of a second stress event. Furthermore, some induced metabolic responses may be effectively permanent or even cumulative, e.g., due to the absence of a catabolic pathway or sequestration mechanism (Mackie et al., 2013). Two phases of metabolic response are evident: (1) metabolic changes that are induced as immediate, specific responses to the stress, e.g., sugar levels, sucrose/hexose ratio, precursors for secondary metabolites or energy-related metabolites. During stress recovery (2) some of these changes may last as mid- or long-term imprints. Imprints may influence upcoming stress responses. Here, we define ***metabolic imprints*** to encompass all metabolic changes that persist after recovery and thus differentiate the imprinted from the initial, pre-stress state.

According to this definition, metabolic imprints are indicators of past environmental conditions and/or stress events. In this sense, metabolic imprints may store information. Imprinted information becomes ***metabolic memory***, when it is maintained and used by the plant system to improve future stress responses, for example to enhance or accelerate metabolite-induced signaling (Thellier and Lüttge, 2013). Metabolic imprints may be caused by one or more environmental events in the individual history of a plant. Metabolic imprints have been postulated previously to act as a stress memory (Bruce et al., 2007). A metabolic memory may act alone or more likely in synergy with priming and memory mechanisms at other system levels that are highlighted in the following.

## Metabolites Influence Biological Systems at all Levels

If metabolites are involved in stress responses and represent stored information, metabolites should in turn influence metabolic or signaling pathways and other parts of plant physiology to modify a stress response. Generally, stress metabolism is seen as hierarchically organized, where external cues initiate signaling pathways that *via* transcription, translation, posttranslational modifications such as phosphorylation and further regulatory steps ultimately affect metabolism. Metabolites are generally thought to represent the downstream “end” products of this hierarchy. Interestingly, this view is currently complemented by findings that suggest bottom-to-top signaling mechanisms. Specific metabolites can exert regulatory influence or feedback on the stress-signaling network and physiology. Such mechanisms open possibilities for cross talk between stress-induced metabolites and other levels of physiological regulation (Bonawitz et al., 2012; Farre and Weise, 2012; Xiao et al., 2012; Gaudinier et al., 2015; Katz et al., 2015; Francisco et al., 2016; Malinovski et al., 2017). Further complexity is indicated by fluxes of central metabolism that are not necessarily explained by transcript abundances of the corresponding enzymes (Chubukov et al., 2013; Schwender et al., 2014). In addition to classical allosteric feedback responses, such as the suppression of enzyme activity by high levels of reaction products, metabolite ratios, and possibly also metabolite fluxes may thus play important roles by directly affecting multiple levels of the stress response hierarchy.

For example, branched chain amino acids are required for phosphorylation of G proteins during osmotic stress signaling in yeast (Shellhammer et al., 2017). Other findings suggest that certain primary metabolites can influence physiology at the transcriptional level. In yeast, Pinson et al. (2009) found that a metabolic intermediate of purine metabolism influences the interaction of transcription factors and thereby modulates purine and phosphate metabolism. Amino acids and polyamines are suggested to directly modify translation because they can be sensed by translating ribosomes *via* interactions with nascent polypeptides, specifically with so-called arrest peptides (Seip and Innis, 2016). Furthermore, several metabolites are cofactors or co-substrates of chromatin-modifying enzymes and thus represent a potential regulatory interface between the metabolic and chromatin states of the cell (Shen et al., 2016; Van der Knaap and Verrijzer, 2016). For some primary metabolites, a role in chromatin modulation is suggested, e.g., fumarate, succinate, α-ketoglutarate, and acetyl-CoA. Fumarate, for example, is a competitive inhibitor of α-ketoglutarate, which is a co-substrate of histone demethylases and TET DNA methylases. Changes in cellular fumarate levels or ratios of fumarate, e.g., to ketoglutarate, may therefore contribute to altered histone modification. Different methylated histone residues are sensitive to changes in the α-ketoglutarate/succinate ratio (Van der Knaap and Verrijzer, 2016). These effects may be specific for certain genetic regions.

The TOR and SnRK kinases are sensors of the cellular energy state and can regulate large parts of metabolism. Plants adapt to changes in energy requirements during stress using these sensors (Baena-González, 2010; Hey et al., 2010; Rexin et al., 2015). The kinases are suggested to respond to sugars and other metabolites, even though the molecular mechanisms are not yet unraveled in all details. TOR is known to be regulated by nutrient sensing of nitrogen and carbon metabolites in plants, yeast, and mammals (e.g., Dobrenel et al., 2016; González and Hall, 2017). But plant defense metabolites of the glucosinolate family, 3-hydroxypropylglucosinolate, and/or its derivatives, can also activate TOR kinases (Malinovski et al., 2017). A precursor of plastidial isoprenoids that is induced by abiotic stress can induce nuclear stress-responsive genes *via* retrograde signaling (Xiao et al., 2012). The mediator complex, which regulates gene transcription, is involved in phenylpropanoid metabolism and is suggested to respond in a feedback loop to changes in this defense-related class of compounds (Gaudinier et al., 2015).

## Metabolites and Stress Responses

Metabolic responses to stress are ubiquitous and well described for many plant systems. Time-series experiments revealed that metabolic activities can respond to stress more quickly than transcriptional activities (Kaplan et al., 2007; Guy et al., 2008; Caldana et al., 2011; Fraire-Velázquez and Balderas-Hernández, 2013), thus making metabolic changes an important part of early stress responses. Several metabolites can directly influence plant stress responses (Rojas et al., 2014). A few important examples are discussed below.

### Carbohydrate Metabolism

During freezing and drought, soluble sugars, such as sucrose, trehalose, fructans (fructose-based oligo- and polysaccharides), and the raffinose family of oligosaccharides can stabilize phospholipid membrane vesicles (Hincha et al., 2006; Livingston et al., 2009; Tarkowski and Van den Ende, 2015). Several sugars emerged as important factors also during biotic stress signaling (Baena-González and Sheen, 2008; Figueroa and Lunn, 2016; Li and Sheen, 2016). Sucrose has been shown to regulate various stress-related responses including circadian clock genes, phytohormones, energy metabolism, cell wall and anthocyanin synthesis (Thibaud et al., 2004; Gomez-Ariza et al., 2007; Tognetti et al., 2013; Tauzin and Giardina, 2014). Glucose induces the pathogen defense proteins PR-1 and PR-5 in *Arabidopsis via* hexokinase1 (HXK1) signaling (Xiao et al., 2000; Moore et al., 2003; Cho et al., 2009). For fructose, a specific pathway has been proposed that involves abscisic acid and ethylene signaling (Cho and Yoo, 2011; Li et al., 2011). Engelsdorf et al. (2013) showed that carbohydrate availability influences defense against a hemibiotrophic fungus during its necrotrophic phase. The more carbohydrate is available, the better the plant defends. Similarly, increased relative fructose content enhances defense against the pathogen *Botrytis cinerea* in tomato (Lecompte et al., 2017). Besides the importance of sugars for stress signaling, plants organize sugar distribution in a way that pathogens have reduced access to carbohydrates. Local depletion of nutrients appears to cause a starvation effect that reduces pathogen propagation (Bezruczyk et al., 2018).

### Amino Acid Pathways

In a 2010 paper, Liu and colleagues knocked out the amino acid transporter lht1 and correlated cellular depletion of the amino acid glutamine with altered redox status and more effective defense against several pathogens (Liu et al., 2010). These authors proposed a yet unknown negative effect of glutamine on defense signaling and a reduction in the pathogen’s access to essential nutrients, similar to recent findings on *Pseudomonas*-primed systemic responses of *Arabidopsis* (Schwachtje et al., 2018). Stuttmann et al. (2011) described threonine as a potential growth inhibitor of the biotrophic oomycete *Hyaloperonospora arabidopsidis*, even though the underlying mechanism is yet unknown. It is suggested that indole-3-carboxylic acid activates defenses against *Plectosphaerella cucumerina* in *Arabidopsis* by inducing papillae deposition and H_2_O_2_ production, independently of salicylic acid and jasmonic acid (Gamir et al., 2014b). γ-Aminobutyric acid (GABA) interacts with quorum sensing of *Agrobacterium tumefaciens*, thus reducing pathogen virulence in tobacco (Chevrot et al., 2006). GABA also functions as a direct anti-herbivore defense in *Arabidopsis* (Scholz et al., 2015). Furthermore, the proline and the pyrroline-5-carboxylate (P5C) cycle are crucial for defense responses against pathogens and abiotic stresses (Liang et al., 2013; Qamar et al., 2015). Proline is involved in redox balance, osmoprotection, and stress signaling (Szabados and Savouré, 2009).

### Polyamine Metabolism

Polyamines (e.g., spermine, spermidine, and putrescine) are aliphatic compounds that are synthesized from amino acids (e.g., arginine and ornithine). Polyamines are involved in many crucial processes of cell metabolism and the translation/transcription machinery, and induce ROS, Ca, and NO signaling (Alcázar et al., 2010). Salt, heat, and drought stress induce genes for polyamine synthesis (Tiburcio et al., 2014; Liu et al., 2015; Miller-Fleming et al., 2015) and enhanced tolerance of abiotic stresses is correlated with elevated levels of polyamines (Alcázar et al., 2010). Putrescine induces abscisic acid synthesis at the transcriptional level during cold stress (Cuevas et al., 2008). Spermine appears to protect *Arabidopsis* from heat stress by increasing the expression of genes encoding heat shock proteins (Sagor et al., 2013). The pretreatment of tomato fruits with spermine before heat shock promoted an increase in expression of signal transduction genes (e.g., calmodulin, serine/threonine protein kinase) along with genes related to phytohormone pathways. Moreover, polyamines can modulate chromatin structure (Pasini et al., 2014). It is also suggested that the connected putrescine, GABA, and proline pathways play an important role during abiotic stresses (Shelp et al., 2012, Legocka et al., 2017).

### External Application of Natural Compounds

A few metabolites have been shown to prime pathogen-induced stress responses when externally applied to plants, e.g., thiamine (Ahn et al., 2007), riboflavin (Zhang et al., 2009), quercetin (Jia et al., 2010), and hexanoic acid (Aranega-Bou et al., 2014). Supposedly, all have in common an activation of the redox system that supports stress signaling. In a recent study, fumarate and citrate applications were shown to induce priming against *Pseudomonas syringae* in *Arabidopsis*, in the case of fumarate without changes in classical defense-related genes and hormones (Balmer et al., 2018). Thereby, Balmer et al. (2018) confirmed an earlier observation of systemic fumarate priming upon first exposure to the bacterial pathogen (Schwachtje et al., 2018). Further, a broad induction of plant defense systems in *Arabidopsis* was demonstrated after the application of melatonin (Weeda et al., 2014).

## Are Stress-Related Metabolites Stored in the Vacuole?

To exert a long-term effect that primes future stress responses, relevant metabolites must be stored in a way that prevents them from negatively interfering with metabolism during the recovery phase and that inhibits degradation. Accumulation of metabolites in chloroplasts, mitochondria, or in the cytosol would likely disturb core metabolic processes that are necessary for recovery (Plaxton, 2005). Possible circumventions would be a reversible conjugation that alters the chemical property of the metabolite, or storage in a membrane-enclosed cellular compartment, such as the vacuole. The vacuole can occupy more than 80% of the cell’s volume and is involved in multiple critical cellular functions including storage of metabolites and modification of cytosolic metabolism according to physiological requirements (Martinoia et al., 2012). The tonoplast enclosing the vacuole contains many identified membrane proteins that are responsible for loading and unloading a diverse set of metabolites (Martinoia et al., 2012). These transporters are integrated in a larger cellular network that responds to physiological requirements and stress responses (Martinoia et al., 2007; Pommerrenig et al., 2018). The carboxylic acids fumarate, malate, and citrate represent major components of the vacuolar metabolome. For example, malate is transported by two proteins, a specific anion channel (Hafke et al., 2003) and a solute carrier (Emmerlich et al., 2003). Many other primary metabolites, such as amino acids and sugars, are stored in the vacuole (Neuhaus, 2007; Tohge et al., 2011; Szekowka et al., 2013; Hedrich et al., 2015). Vacuoles are also involved in plant defenses against herbivores and pathogens by storing and sequestering toxic metabolites. Thus, specific analyses of vacuolar metabolite compositions, e.g., by non-aqueous fractionation (Klie et al., 2011), are required to unravel a possible long-term storage of stress-induced metabolites that represent metabolic imprints and may function as a metabolic memory.

## Examples of Stress-Induced Metabolic Imprints

The functional analysis of stress-induced metabolic responses has been a long-standing focus of plant physiology. In contrast, metabolic imprints following stress have rarely received attention but can now be described and analyzed in detail by large-scale experiments that combine metabolic phenotyping with global screening of other system levels and physiological analyses (e.g., Hemme et al., 2014). The fragmented knowledge on recovery processes may result from the simplifying assumption that plants will revert to the identical initial endogenous state after a transient environmental perturbation. The resetting of the initial state is thought to alle*via*te the need to expend energy for the maintenance of the stress-adapted state. While this assumption may be correct for the majority of metabolites, the past perturbations may leave an imprint on metabolism that lasts longer than may be expected from a system level that is notorious for its extremely rapid fluctuations (Urbanczyk-Wochniak et al., 2005; Kim et al., 2011). In the following, we will review evidence of metabolic imprinting and functions of imprints for priming of systems in the context of abiotic and biotic stresses.

### Abiotic Stress

Abiotic stresses are known to prime plant systems for an enhanced stress response to a recurrent stress. Recent reviews highlight abiotic stress priming of temperature, drought, and other factors (Bruce et al., 2007; Hincha and Zuther, 2014; Hilker et al., 2015). In the following, we will first highlight proline imprints that were observed in the context of various stresses before we address more stress-specific metabolic imprints.

### Proline Imprints Are Caused by Various Abiotic Stresses

Proline accumulation is one of the most studied metabolic stress responses. Upon environmental stress, proline is mainly generated from glutamic acid in chloroplasts and increases up to 100-fold in plants (Liang et al., 2013). Proline has several functions during stress responses, e.g., as an osmoprotectant, antioxidant, molecular chaperone to protect protein integrity, pH buffer, or in some cases it may serve as a carbon and nitrogen source during stress recovery. Proline also enhances enzyme activities, triggers gene expression, and modulates mitochondrial functions (Szabados and Savouré, 2009). By increasing ROS production in mitochondria *via* the electron transport chain, proline regulates processes that support cell survival or induce apoptosis (Liang et al., 2013). Suppression of proline catabolism, for example *via* reduction of proline dehydrogenase gene expression, enhances tolerance toward salt and drought stress (Ibragimova et al., 2012). In *Arabidopsis*, proline accumulates strongly during a 4-day drought phase and declines to initial levels during a subsequent 4-day recovery phase (Sharma and Verslues, 2010). In contrast, the drought-resistant *Periploca sepium* increases proline levels continuously during a similar 4-day drought stress but maintains a proline imprint during a 4-day recovery phase (An et al., 2013). Even after a 8-day recovery, the newly developed buds of *Periploca sepium* still contained twice as much proline as control plants. Proline is apparently also important for recovery of tobacco plants from drought stress by suppressing a senescence-related promoter (Vancova et al., 2012). In addition, the proline concentration in salt stress-resistant salt cress (*Thellungiella halophila,* renamed to *Thellungiella salsuginea,* and *Eutrema salsugineum*) is significantly higher than in *Arabidopsis* already under control conditions (Taji et al., 2004; Benina et al., 2013; Lee et al., 2016). In this case, high proline levels may serve as a constitutive stress adaptation of an extremophile plant. Also, in *T. halophila*, proline levels increased during a 3-day recovery from cold stress but not during the 3-day stress phase itself. The imprint of the proline pool is accompanied by other metabolites, such as 5-hydroxyproline and sucrose (Benina et al., 2013). In *Arabidopsis*, 3 days after de-acclimation from cold acclimation, proline levels were still elevated in leaves. More freeze-tolerant *Arabidopsis* accessions showed higher levels than susceptible accessions after 3 days of recovery (Zuther et al., 2015).

### Drought

Several studies describe imprints of metabolite pools other than proline after exposure to drought. Primary metabolites, e.g., sugars and organic acids, as well as several secondary metabolites maintain a characteristic imprint in the resurrection plant *Haberlea rhodopensis* after 2 days of recovery from an 8-day drought period (Moyankova et al., 2014). A similar duration of drought stress and recovery, 8–10-day stress and 2-day recovery, causes a different metabolic imprint of *Medicago sativa* nodules (Naya et al., 2007). In this symbiotic system, pools of several primary metabolites remain reduced during drought recovery. A recent study describes the metabolic recovery of drought-stressed sugar beets (Wedeking et al., 2018). The authors found a transient normalization of most of the measured metabolites after 8 days of recovery from a 13-day drought stress period. Interestingly, during the following 4 days, several amino acids (e.g., phenylalanine, tyrosine, and leucine) again accumulated in leaves, indicating a metabolic stress imprint that may be beneficial for a subsequent second drought phase.

### Low Temperature

Faster than drought stress, temperature stress may rapidly revert. Temperature changes cause metabolic responses that are particularly well characterized after heat shock or extended cold exposure (e.g., Kaplan et al., 2004, 2007; Guy et al., 2008). The temperature-induced metabolic responses comprise strong changes in a wide range of metabolite pools that indicate global reprogramming of primary metabolism in *Arabidopsis* rosettes. Data on metabolomic and transcriptomic cold recovery describe a 24-h metabolic imprint after 4 days of exposure to 4°C (Kaplan et al., 2004, 2007) and reveal several interesting aspects. Firstly, most of the cold-induced transcript changes returned to the pre-stress state after 24 h. In contrast, primary metabolism was only partially recovered. These differences between transcriptional and metabolic recovery from cold stress were recently confirmed in greater detail by Pagter et al. (2017). Secondly, the metabolite profile of the recovery phase differed significantly from all measured time points during cold exposure, supporting the observation that the metabolic reorganization after stress exhibits different kinetics than the stress response. Cold de-acclimating metabolism was associated with partially maintained enhanced freeze tolerance, which apparently remains active at least 3 days into cold-recovery (Kaplan et al., 2004; Zuther et al., 2015). Zuther et al. (2018) reported genetic differences in the transcriptomic and metabolic patterns during cold memory of the *Arabidopsis* ecotypes, Col-0 and N14.

A complex picture of metabolic reorganization during recovery was described after freezing stress of crown tissue of oat (*Avena sativa* L.) by Henson et al. (2014). After 3 weeks of cold acclimation and 1 day of freezing, plants were monitored during 14 days of recovery. At the end of recovery, several amino acids were largely increased compared to non-stressed plants, and several sugars and organic acids were reduced. Moreover, the metabolic profile differed markedly from what is observed after cold stress recovery, indicating that this overwintering species relies on specific regulations for freezing resistance.

Analyses of *Hordeum vulgare* also show stress-imprinted metabolites that are linked to frost tolerance (Mazucotelli et al., 2006). For example, 8-day-old barley seedlings were freeze-stressed at −3°C for 16 h and allowed to recover for 48 h at 22°C. This treatment resulted in 16-fold higher GABA levels at the end of the recovery phase. GABA and its precursor glutamate are part of the GABA-shunt that is linked to the tricarboxylic acid cycle where it bypasses two reaction steps from α-ketoglutarate to succinate. Besides glutamate, putrescine and proline can also be catabolized *via* GABA (Shelp et al., 2012; Signorelli et al., 2015). The GABA-shunt has a central role in carbon/nitrogen metabolism and stress signaling, for example for cell death promotion in response to pathogens or for cold tolerance (Mazucotelli et al., 2006; Fait et al., 2007; Michaeli and Fromm, 2015). However, the role of GABA during cold−/frost-stress and possible GABA pool imprints are still not fully understood.

### High Temperature

Elevated temperatures leave metabolic imprints in photosynthetic microorganisms. A recent large-scale study describes the temporal succession of heat stress responses of *Chlamydomonas reinhardtii* during a 24-h induction phase after shift from 25 to 42°C and the fate of system imprints during 8-h recovery at 25°C (Hemme et al., 2014). In this experiment, cell division stopped during heat treatment and remained so during the 8 h of recovery, resulting in measurements that represent the metabolic state of cells that all individually experienced the heat stress. Similar to the example of 4°C cold stress in *Arabidopsis* (Kaplan et al., 2004, Pagter et al., 2017), the metabolome, as well as the proteome, recovered only in part and retained imprints, regarding, e.g., TCA intermediates and sugar phosphates. Importantly, the pattern of metabolic induction again differed from the pattern of metabolic recovery.

Besides temperature perturbations, other abiotic stresses have been shown to generate lasting imprints. A 6-h oxidative stress that was induced by menadione generated an imprint on primary metabolism in *Arabidopsis* roots that lasted at least 30 h into recovery (Lehmann et al., 2012). GABA was part of this imprint, like proline and other amino acids which remained at a high level, as well as several sugars and sugar phosphates.

### Biotic Stress

Biotic stresses are perhaps the best understood stresses regarding primed plant systems. Recent reviews highlight the importance of biotic stress priming for enhanced responses toward a broad range of insects and pathogens that negatively influence plant performance and crop production (Frost et al., 2008; Conrath et al., 2015; Hilker et al., 2015; Mauch-Mani et al., 2017). At the metabolite level, biotic priming is mainly studied with respect to volatile organic compounds, oviposition, and beneficial or pathogenic microorganisms that are associated with a plant and may prime systemic tissue. Even though it has often been shown that during biotic stresses, metabolism in local and systemic plant parts is severely affected (Schwachtje and Baldwin, 2006; Lemoine et al., 2013; Zhou et al., 2015), studies on persistent metabolic changes during and after recovery from pathogen or insect stress are rare. This applies specifically for interactions of plants with microorganisms, since these are continuously associated with the plant, either as beneficial root colonizers or as leaf pathogens, thus making a clearly defined recovery phase after a time-limited stress or induction phase unfeasible. Nevertheless, several metabolites have so far been associated with priming against biotic stresses.

Plant amino acid metabolism is well known to contribute to the priming of defense responses (Gamir et al., 2014a). For example, the lysine catabolite pipecolic acid can act as a key regulator of SAR (Návarová et al., 2012; Zeier, 2013). Several amino acids and intermediates of the TCA cycle are regulated during priming with pathogenic *Pseudomonas syringae* or the chemical β-aminobutyric acid, i.e., BABA (Pastor et al., 2014). The content of most amino acids was reduced in these experiments, but cysteine, methionine, tryptophan, and tyrosine were specifically induced by bacteria or BABA during 48 h. Fumarate and malate were induced by BABA. These two organic acids were also induced in another study that investigated systemic priming-related effects of infection of *Arabidopsis* with *Pseudomonas syringae* (Schwachtje et al., 2018). A systemic increase of fumarate and malate was observed for 4 days, whereas the transcriptional profile did not explain the altered metabolite levels. This study suggested a lasting metabolic priming effect in systemic tissue that includes storage of metabolites, e.g., fumarate and malate. These metabolites may be readily available to support energy and carbon demands during a subsequent pathogen (*Pseudomonas syringae*) infection.

Altered amino acid levels after contact with a pathogen can have multiple and possibly conflicting functions. Several amino acids are precursors of important defense metabolites, e.g., alkaloids, phenylpropanoids, and glucosinolates. On the other hand, invasive pathogens like *Pseudomonas syringae* propagate in the apoplast and are exclusively dependent on extracellular plant metabolites. Reduction of sugars and amino acids in the apoplast and, as indicated by transcript changes, likely also other nitrogen resources should be an effective defense strategy that attenuates pathogen propagation and thereby increases the efficiency of other defense mechanisms (Seifi et al., 2013; Bezruczyk et al., 2018; Schwachtje et al., 2018). Several imprinted metabolic signals have been identified that may contribute to the modulation of SAR, including a glycerol-3-phosphate-dependent yet non-identified signal, azelaic acid, dehydroabietinal, jasmonic acid, and methyl salicylate (Dempsey and Klessig, 2012). The metabolic signals that are linked to SAR or other primed responses will yield intriguing novel insights into imprinted primary metabolism/energy status and the function of such imprints for efficiently primed plant responses.

In their natural environment, plants usually face more than one type of stress. The physiological responses toward various stress combinations, simultaneous or successive, have been addressed by recent studies (reviewed in Suzuki et al., 2014). The effects on plant performance can be synergistic, neutral, or conflicting (Crisp et al., 2016; Lawas et al., 2018) and it will be a demanding task to unravel how metabolite-based priming and priming in general by a certain stress may influence plant responses to other types of stress.

## Experimental Approaches

The successful search for priming-related metabolites relies on the timing of experiments. Mostly, stress studies focus on the immediate response of the plant system toward an applied stress, but rarely focus on the long-term effects on plant metabolism. The recovery phase after a stress event is crucial for the establishment of priming and should thus be studied more extensively. The history of plants prior to stress experiments is rarely controlled and comparable between experiments. These methodology details must be described in detail to enhance reproducibility of stress experiments.

Several factors interfere with the metabolic state of a plant during the recovery phase and must be experimentally addressed. As described above, the metabolic composition of plant tissues is an integral of perceived environmental stresses (Gratani, 2014) and may lead to variation among individual plants even under standardized conditions (e.g., Sanchez et al., 2010). Metabolism is continuously regulated by the circadian clock (Farre and Weise, 2012) and this regulation also affects stress responses themselves on genetic and metabolic levels (Lu et al., 2017). For example, glucosinolate accumulation follows the circadian rhythm in *Arabidopsis* and jasmonic acid-based defenses are synchronized with the likeliness of herbivore attack (Goodspeed et al., 2012). In return, several biotic stressors have recently been shown to influence the cycle length of the circadian clock, e.g., pathogens and insects (Sharma and Bhatt, 2015; Joo et al., 2018; Li et al., 2018). This requires experimental setups with extended sampling time points during the day. Also, the influence of the ontogenetic stage, i.e., the effects of endogenous physiological aging mechanisms, on induced metabolic responses should be addressed. Furthermore, as described above, priming-related metabolites may be stored in certain cell compartments (e.g., the vacuole). Subcellular localization of metabolites is difficult to assess but can be addressed by non-aqueous fractionation (Klie et al., 2011). Because temporal effects are essential for the assessment of induced, imprinted, and primed responses, care should be taken to design time-series experiments with extended and high temporal resolution including the coverage of diurnal changes. High replication is advised due to varying histories and developmental variation of individual plants (e.g., Peters et al., 2018), this applies particularly to field experiments. Further, the high chemodiversity of plant metabolites entails many different chemical properties. To find new candidates for priming, the application of multiple chromatography systems for untargeted metabolic profiling should be taken into account (e.g., Nakabayshi and Saito, 2015; Vasilev et al., 2016).

## Conclusion

Recent publications tackle the study of metabolic imprints by analyses of recurrent perturbations or recovering plant systems and discover functions of novel primed metabolites and metabolic pathways (Gamir et al., 2014a; Balmer et al., 2015). Intensified research on the potential functions of metabolic imprints should be highly fruitful and yield novel insights into priming phenomena. This view is supported by recent findings that demonstrate surprisingly diverse effects of metabolites on stress metabolism, signaling, and transcription. The vast chemical diversity of plants will likely yield new candidates of metabolic regulation or priming.

Currently, the knowledge of the short- to long-term kinetics of metabolic imprints is fragmented. This fact renders vague the link between observed metabolic imprints and their potential function as priming signals or memory of past stress events. Except for the known signaling metabolites that are involved in primed plant responses, the nature, characteristics, and role of metabolic imprinting or priming remain mostly unknown not least because stress recovery is rarely investigated in depth by studies employing modern large-scale metabolomic, proteomic, transcriptomic, or epigenetic tools. From advanced analyses of metabolic imprints, we expect to discover new priming mechanisms and to gain insight into the major contributions of metabolism to priming and potentially short-lived or even longer-lasting non-neural, cellular memory.

## Author Contributions

JS and JK developed the concepts and wrote the manuscript with contributions of all other co-authors.

### Conflict of Interest Statement

The authors declare that the research was conducted in the absence of any commercial or financial relationships that could be construed as a potential conflict of interest.
